# Collaboration, campaigns and champions for appropriate imaging: feedback from the Zagreb workshop

**DOI:** 10.1007/s13244-018-0602-9

**Published:** 2018-03-12

**Authors:** D. Remedios, B. Brkljacic, S. Ebdon-Jackson, M. Hierath, V. Sinitsyn, J. Vassileva

**Affiliations:** 1 Department of Clinical Radiology, Northwick Park Hospital, Harrow, UK; 2 Department of Diagnostic and Interventional Radiology, University Hospital Dubrava, University of Zagreb School of Medicine, Zagreb, Croatia; 3 Directorate CRCE, Public Health England, Chilton, Didcot, Oxfordshire UK; 4 Department of European and International Affairs, European Society of Radiology, Vienna, Austria; 5 Department of Radiology, Federal Center of Treatment and Rehabilitation, Moscow, Russian Federation; 6 International Atomic Energy Agency, Vienna International Centre, Vienna, Austria

**Keywords:** Radiology, Justification, Appropriate imaging, Radiation protection, Imaging referral guidelines

## Abstract

**Abstract:**

Leading radiologists and representatives from national radiation protection regulatory authorities and health ministries from 19 countries of the European region worked together with five experts at the workshop on justification and appropriate use of imaging in Zagreb, Croatia, from 26 to 28 October 2017 jointly organised by the IAEA and the European Society of Radiology. The workshop served as a forum to exchange information on challenges and solutions for improving justification and the appropriate use of diagnostic imaging. Common barriers to improving the use of imaging referral guidelines were discussed and the need for increased collaboration identified. Examples of good practices were presented, including use of Clinical Decision Support (CDS) systems to facilitate rapid and good justification decisions. The workshop identified some of the needs of European countries for achieving more appropriate imaging proposing wider use of *collaboration, campaigns and champions.*

**Main messages:**

*• Drivers for appropriate imaging in Europe are similar to those elsewhere globally.*

*• Implementing imaging referral guidelines is the main barrier to more appropriate imaging.*

*• Clinical Decision Support systems (CDS) facilitates good referral practice and justification decisions.*

*• Collaboration, campaigns and champions may improve awareness, appropriateness and audit.*

## Introduction

Justification and appropriate imaging have been key topics in global efforts pulling together drivers from evidence-based practice, radiation protection and value-based imaging [1–7]. Regional efforts in Africa, the Middle East, South East Asia, Latin America and the Far East [8–13] have complemented more established efforts in North America [14, 15] and Western Europe [16]. The wish to consolidate efforts in some countries, particularly in the Eastern European region, led to the organisation of the Zagreb workshop from 26 to 28 October 2017. This regional workshop was held in Zagreb, Croatia, by the International Atomic Energy Agency (IAEA), co-sponsored by the Government of the Republic of Croatia, City of Zagreb, and the European Society of Radiology (ESR), and supported by the World Health Organisation (WHO). Leading radiologists and representatives from national radiation protection regulatory authorities and health ministries were invited, with 40 participants from 19 countries working with 5 experts. Common barriers to appropriate imaging of patients and asymptomatic individuals were identified with good practices shared and collaborative solutions sought (Tables 1).

## Common barriers, suggested solutions and good practices

Participants identified common barriers and solutions for more appropriate imaging of patients and asymptomatic individuals not referred by a doctor, giving useful examples from their local practices (see Tables 2 and 3). All participants pointed out that imaging referral guidelines were not officially used in their countries. The most common barrier was the difficulty in implementing imaging referral guidelines and tools for better use. Some countries had managed to provide the distribution and some promotion of imaging referral guidance. For example, in Poland 100,000 copies of the adopted and translated guidelines have been distributed. Several institutions in Croatia, Russia and Sweden have piloted Clinical Decision Support systems (CDS) bringing guidance closer to referrers. Use of CDS systems such as the ESR iGuide [1] and RCR iRefer [3] will rapidly facilitate good referral practice and justification decisions through adopting, adapting and translating where needed.

**Table 1:** Participating countries for the Zagreb Workshop

EU countries	Non-EU countries
Bulgaria	Armenia
Croatia	Bosnia and Herzegovina
Estonia	Georgia
Lithuania	Kazakhstan
Latvia	FYR Macedonia
Poland	Montenegro
Portugal	Moldova
Romania	Russia
UK	Turkey
	Ukraine

**Table 2:** Areas of difficulty and solutions for providing more appropriate imaging for patients

Area of difficulty	Need
Imaging referral guidelines	Up-to-date, evidence-based referral guidelines and tools for use Better if a regulatory requirement Choice will depend on healthcare practice, technology and economy Paper, web, app and CDS versions available
Justification for patients	Good regulations. Responsibilities should be specified and taken
Professional and legal support	Common professional and regulatory support for the correct examination at the right time, based on the clinical question and information provided
Education and training	Education and training according to national needs Targeted to all stakeholders as required and at all levels including CPD Escalate if needed Workflow solutions
Gatekeeper role	Radiologist should have the time and authority to amend or return requests

**Table 3:** Issues to consider for more appropriate imaging in asymptomatic individuals not referred by a doctor

Issues	Need
Guidance	Guidance must be evidence-based especially for population screening and individual health assessment (IHA) for asymptomatic individuals
Individual Health Assessment of asymptomatic individuals (IHA)	For asymptomatic individuals IHA, risk factors should be used to replace symptoms to inform justification If symptomatic then should re-enter justification pathway for patients Data from IHA should inform healthcare population studies
Research ethics	Medical Physics Experts and Radiologists are needed for ethical committee decisions in research
Conflicts of interest	Conflicts of interest must be managed to avoid harm to individuals
Ethical principles	Ethical considerations needed for immigration, employment, sports imaging

Many smaller countries reported the difficulty with resources, both human and financial. Training and educational needs were also common. Portugal provided a good example of collaboration through a common language. With eight Portuguese-speaking countries in four regions (Angola, Brazil, Cabo Verde, Guinea Bissau, Mozambique, Portugal, San Tome et Principe and Timor-Leste), efforts for radiation safety, the burden of cost and value of benefits are shared. Plans have also been made for collaboration through the Russian language in several Eastern European Region countries.

Clinician awareness, acceptance and co-operation posed a problem in several countries. Workflow solutions such as multi-disciplinary clinical meetings enabled better communication and education of clinician colleagues in the Baltic States.

In some countries outside the European Union there was a wish for stronger and transparent legislation to provide a regulatory framework and means for inspection. This was particularly apparent in countries where the culture was for referrers to *order* rather than *request* investigations, losing the opportunity for radiologists’ valuable input as to the most appropriate imaging investigation, if any is needed. The need for a radiological practitioner as gatekeeper was clear. The under-provision of equipment in some countries precluded the use of the best test first but it was accepted that in these health economies latitude and flexibility should be exercised correctly.

## Discussion

In Europe as in other regions of the world, there are common needs for better justification of medical exposures leading to more appropriate imaging, less waste and more sustainable use of imaging resources [17]. The same drivers for appropriate imaging are apparent in Europe as in Africa, America and Asia. The inequity and iniquity of over-utilisation and under-utilisation of radiology within the same jurisdiction exist in most countries with 20–45% of examinations deemed inappropriate [18]. The nirvana of uniform requesting, use and provision of imaging will need different approaches in different countries [19].

Examples of good practices and the wish for collaborative efforts identified several common areas for progress (Table 4). It was clear that the need for greater collaboration would provide benefits faster, sharing the burden of work. Initiatives that have already been taken up or will be shortly are:

- Collaboration through a common professional language.
- Collaboration geographically by neighbouring countries.
- Collaboration of countries sharing similar culture and/or ethnicity.
- Collaboration through a similar technology, e.g. CDS, for requesting appropriate imaging investigations.
- Champions for good practice within and among countries.
- Campaigns to promote awareness and good practices.

**Table 4:** Suggestions for the next steps forward

Initiative	Examples
National/local implementation of justification tools	1. Adopting / adapting Imaging Referral Guidelines from a trusted source (professional societies, regulatory competent authorities) 2. Support from regulators, health ministries, medical societies radiological and clinical, radiographic societies, medical physicists 3. Public awareness for expectations and safety, e.g. EuroSafe Imaging and Choosing Wisely campaigns [16, 20, 21]
Education/training	1. Training material from professional societies and international / national organisations, e.g. IAEA, WHO, ESR, RCR, ACR, RANZCR etc. 2. Radiation protection training within university curricula, education in workflow, continuing professional development 3. Regional/sub-regional/national training workshops—IAEA, WHO, ESR
Feedback by the IAEA, WHO	1. National radiation protection competent authorities and 2. Ministries of Health

Abbreviations: IAEA, International Atomic Energy Agency; WHO, World Health Organisation; ESR, European Society of Radiology; RCR, UK Royal College of Radiologists; ACR, American College of Radiology; RANZCR, Royal Australian and New Zealand College of Radiologists

## Conclusion

The needs of European countries for achieving more appropriate imaging are becoming clearer and concise. The putative principles of *collaboration, campaigns and champions* may be the way forward for the accepted axiom of *awareness, appropriateness and audit*.
